# Supplementary material for: An application of COM-b model to explore factors influencing veterinarians’ antimicrobial prescription behaviors: Findings from a qualitative study in Bangladesh
Source: PLoS One. 2024 Dec 16;19(12):e0315246. doi: 10.1371/journal.pone.0315246 (PMC11649135; doi:10.1371/journal.pone.0315246)
Supplement: Attachment — Submitted filename: Response to reviewers.docx [file pone.0315246.s003.docx]

When submitting your revision, we need you to address these additional requirements.

1. Please ensure that your manuscript meets PLOS ONE's style requirements, including those for file naming. The PLOS ONE style templates can be found at 
<https://journals.plos.org/plosone/s/file?id=wjVg/PLOSOne_formatting_sample_main_body.pdf> and 
<https://journals.plos.org/plosone/s/file?id=ba62/PLOSOne_formatting_sample_title_authors_affiliations.pdf>

Response: We have adhered to PLOS’s style requirements. Please check in the revised version.

2. Thank you for submitting the above manuscript to PLOS ONE. During our internal evaluation of the manuscript, we found significant text overlap between your submission and previous work in the [introduction, conclusion, etc.].
 
We would like to make you aware that copying extracts from previous publications, especially outside the methods section, word-for-word is unacceptable. In addition, the reproduction of text from published reports has implications for the copyright that may apply to the publications.
 
Please revise the manuscript to rephrase the duplicated text, cite your sources, and provide details as to how the current manuscript advances on previous work. Please note that further consideration is dependent on the submission of a manuscript that addresses these concerns about the overlap in text with published work.
 
[If the overlap is with the authors’ own works: Moreover, upon submission, authors must confirm that the manuscript, or any related manuscript, is not currently under consideration or accepted elsewhere. If related work has been submitted to PLOS ONE or elsewhere, authors must include a copy with the submitted article. Reviewers will be asked to comment on the overlap between related submissions (<http://journals.plos.org/plosone/s/submission-guidelines#loc-related-manuscripts)>.]
 
We will carefully review your manuscript upon resubmission and further consideration of the manuscript is dependent on the text overlap being addressed in full. Please ensure that your revision is thorough as failure to address the concerns to our satisfaction may result in your submission not being considered further.

Response: This comment was withdrawn by the journal on February 09 via email. However, we have revised the introduction and recommendation sections, although these modifications do not substantially alter the narrative of the manuscript.

3. Thank you for stating the following in the Acknowledgments Section of your manuscript: 
"We would like to thank the research participants who gave their valuable time and took participate in the study voluntarily. We also acknowledge the support from Chattogram Veterinary and Animal Science University in granting ethical approval of this study. We also grateful to Bangladesh Bureau of Educational Information and Statistics (BANBEIS) for providing partial funding (ID#SD2019967) to this study. We also acknowledge the contribution of Irina Bergenfeld (a PhD Student in Global Health and Development Program, Emory University, Atlanta, Georgia, USA) for her time and effort to proofread the manuscript. The authors acknowledge with particular gratitude the anonymous reviewers who offered detailed and helpful comments on the manuscript."

We note that you have provided funding information that is not currently declared in your Funding Statement. However, funding information should not appear in the Acknowledgments section or other areas of your manuscript. We will only publish funding information present in the Funding Statement section of the online submission form. 
Please remove any funding-related text from the manuscript and let us know how you would like to update your Funding Statement. Currently, your Funding Statement reads as follows: 
"This work was funded by Bangladesh Bureau of Educational Information and Statistics (BANBEIS), ID number SD2019967. Mohammad Mahmudul Hassan was supported by BANBEIS. The funders had no role in study design, data collection and analysis, decision to publish, or preparation of the manuscript."

Please include your amended statements within your cover letter; we will change the online submission form on your behalf.

Response: We have dropped out the funding information from the acknowledgement section of the revised manuscript (Please check line number 770-780 in the revised manuscript). Please see the funding statement below:

"This work was funded by Bangladesh Bureau of Educational Information and Statistics (BANBEIS), ID number SD2019967. The funders had no role in study design, data collection and analysis, decision to publish, or preparation of the manuscript."

4. Thank you for stating the following in your Competing Interests section:  
"NO authors have competing interests."

Please complete your Competing Interests on the online submission form to state any Competing Interests. If you have no competing interests, please state "The authors have declared that no competing interests exist.", as detailed online in our guide for authors at <http://journals.plos.org/plosone/s/submit-now> 
 This information should be included in your cover letter; we will change the online submission form on your behalf.

Response: Please see our Competing Interests below:

"The authors have declared that no competing interests exist."

5. We note that you have indicated that data from this study are available upon request. PLOS only allows data to be available upon request if there are legal or ethical restrictions on sharing data publicly. For more information on unacceptable data access restrictions, please see <http://journals.plos.org/plosone/s/data-availability#loc-unacceptable-data-access-restrictions>.

In your revised cover letter, please address the following prompts:

a) If there are ethical or legal restrictions on sharing a de-identified data set, please explain them in detail (e.g., data contain potentially sensitive information, data are owned by a third-party organization, etc.) and who has imposed them (e.g., an ethics committee). Please also provide contact information for a data access committee, ethics committee, or other institutional body to which data requests may be sent.

b) If there are no restrictions, please upload the minimal anonymized data set necessary to replicate your study findings as either Supporting Information files or to a stable, public repository and provide us with the relevant URLs, DOIs, or accession numbers. For a list of acceptable repositories, please see <http://journals.plos.org/plosone/s/data-availability#loc-recommended-repositories>.

We will update your Data Availability statement on your behalf to reflect the information you provide.

Response: Please find our updated data availability statement below:

Dataset is not publicly shareable because the participants of the study did not give consent to make their interview transcripts available in the public domain. Additionally, the transcript may contain some sensitive information such as the brand name of the antimicrobials, as well as participants could be identifiable because of their professional affiliation, which may put them at risk. However, data request can be made to the corresponding author at mkalam@emory.edu or to the ethical committee directly at drecvasu@gmail.com. An anonymized dataset can be available with an appropriate purpose.

6. Please include captions for your Supporting Information files at the end of your manuscript, and update any in-text citations to match accordingly. Please see our Supporting Information guidelines for more information: <http://journals.plos.org/plosone/s/supporting-information>.

Response: We have added Supporting Information file at the end of the manuscript. We also cited this document within the text.

[Note: HTML markup is below. Please do not edit.]

Reviewers' comments:

Reviewer's Responses to Questions

**Comments to the Author**

1. Is the manuscript technically sound, and do the data support the conclusions?

The manuscript must describe a technically sound piece of scientific research with data that supports the conclusions. Experiments must have been conducted rigorously, with appropriate controls, replication, and sample sizes. The conclusions must be drawn appropriately based on the data presented.

Reviewer #1: Yes

Reviewer #2: Partly

2. Has the statistical analysis been performed appropriately and rigorously?

Reviewer #1: N/A

Reviewer #2: N/A

3. Have the authors made all data underlying the findings in their manuscript fully available?

The [PLOS Data policy](http://www.plosone.org/static/policies.action#sharing) requires authors to make all data underlying the findings described in their manuscript fully available without restriction, with rare exception (please refer to the Data Availability Statement in the manuscript PDF file). The data should be provided as part of the manuscript or its supporting information, or deposited to a public repository. For example, in addition to summary statistics, the data points behind means, medians and variance measures should be available. If there are restrictions on publicly sharing data—e.g. participant privacy or use of data from a third party—those must be specified.

Reviewer #1: No

Reviewer #2: No

4. Is the manuscript presented in an intelligible fashion and written in standard English?

PLOS ONE does not copyedit accepted manuscripts, so the language in submitted articles must be clear, correct, and unambiguous. Any typographical or grammatical errors should be corrected at revision, so please note any specific errors here.

Reviewer #1: Yes

Reviewer #2: Yes

5. Review Comments to the Author

Please use the space provided to explain your answers to the questions above. You may also include additional comments for the author, including concerns about dual publication, research ethics, or publication ethics. (Please upload your review as an attachment if it exceeds 20,000 characters)

Reviewer #1: Dear Author(s)/Editor(s),

Comment: This is a well-written paper with interesting findings. Antimicrobial stewardship is indeed an essential strategy in addressing AMR and veterinarians play a key role in this endeavor. To this end, the application of comprehensive and theory driven behavioral science frameworks such as the COM-B model are both welcomed and necessary. Application of these approaches is important as it highlights factors beyond “knowledge deficits” that pattern antimicrobial use patterns and strives for a more holistic approach beyond the common training strategies focused on awareness raising. To this end, the authors should be commended for taking a behavioral science approach towards understanding veterinary prescription factors. As they correctly note, veterinarians play a key role in determining the types of antimicrobials used and whether these antimicrobials are correctly administered. This is especially the case in LMICs, as regulatory frameworks and guidance are limited, resulting in veterinarians drawing upon on their own experiences to dispense antimicrobials and often in competition with “quacks”, who may not have the background to prescribe the correct antimicrobial. Given the essential role played by veterinarians in animal health, and by extension AMR, the results presented have the potential to make a significant contribution to the field. With this said, we suggest a few major and several minor revisions that should be considered before publication. We also attach a pdf with minor comments.

Response: Thanks so much for your time and enthusiasm to read the manuscript and provided valuable feedback.

Major Revisions

Comment: We would suggest that the findings be clustered more carefully into the behavioral science frameworks. There were a few instances where it was difficult to see how certain codes were clustered into a particular domain, especially when there was ambiguity. A stronger justification for these clustering decisions would be helpful (specific instances with corresponding line numbers found in “Minor Revisions”).

Response: Thanks so much for your comment. As we mentioned in the draft, we used the COM-B model as our guiding principle to carry out our study and our analysis was built on both inductive and deductive coding. Hence, the decision for clustering was done by matching the text segments and the nature of the inductive codes. Before making the final decision on the case of ambiguity, we took two strategies. Firstly, consulting with the literature and secondly, consensus among the coders. It is important to note here, since there is lack of studies available on the use of COM-B model in studying the behavioral determinants of antimicrobial prescription by the veterinarians, we consulted with other research areas such as prescription of antibiotics (and other medicines) for human use, and other health issues. To achieve the consensus among coders, the coders discussed among themselves and made the final decision. Please see related information in line number 250-260 in the revised paper.

Comment: The recommendations focus heavily on educational interventions, with relatively little attention given to interventions addressing motivation. It would be helpful to provide a more balanced approach to the recommendations, given that motivation is a critical factor in the COM-B.

Response: Thanks so much for your comment. We have now added text on other areas of recommendations including strategies to increase motivation. Please see the revised version throughout which starts with policy priority to make the testing facility available first, followed by increase the sense of shared responsibilities through collective efforts and increase the motivation and other domains of the intervention based on the findings of different domains of COM-B model.

Comment: The data analysis section could be clarified to better explain how the codes were clustered into the frameworks and how COM-B was combined with the TDF. Providing more specific examples to illustrate your points would also be helpful. (Again, specific instances with corresponding line numbers found in “Minor Revisions”)

Response: Thanks so much for your comment. As we mentioned in our earlier response, we first developed the deductive codes based on the domains of the COM-B model and the data collection guideline. We developed the inductive codes based on what the research participants responded. Then these codes were categorized and clustered based on the attributes, (definitions and applicability) of the codes and the domains of the COM-B model. This approach has been described in the book titled “Qualitative Research Methods” – by Hennink, Hutter and Bailey. However, the combination of COM-B model and the TDF was made based on the similarities of each domain. In doing so, we grouped the sub-domains of TDF under different domains of COM-B model, as done by Annemarie De Leo and colleagues in their 2021 study (PMID 33436092). De Leo et al. was referred and cited in the paper (Figure 2 in that paper for your reference). Table 2 in the original draft has presented this strategy.

Comment: Finally, the paper does not investigate cultural and psychological factors in depth. These factors can have a significant impact on behavior, and it would be helpful to consider them more fully in the analysis and discussion or possibly as a recommendation for Future Research.

Response: Thanks so much for your observation. For us, the domain “Opportunity” broadly covers cultural factors. Especially, the sub-domain titled “Social Influence” captured many of the aspects of cultural factors of antimicrobial prescriptions. As the original article by Michie et al mentioned “the environment and social opportunity afforded by the cultural milieu that dictates the way that we think about things (e.g., the words and concepts that make up our language).” [PMID: 21513547 or <https://doi.org/10.1186/1748-5908-6-42>] However, we do agree that the socio-cultural properties around antimicrobials were not captured in-depth, in part due to the adopting the COM-B framework.

However, for the psychological factors, our findings on psychological capability covers the factors that COM-B model suggests capturing. Specifically, we have captured psychological factors like knowledge, decision process and behavioral regulation. Our findings under the sub-theme “Knowledge on AMR”, “Knowledge about reserve group of antimicrobials”, “ability to differentiate groups of antimicrobials and their use” and “factors influencing choice of antimicrobials" covered these psychological factors.

Minor revisions (Line Number preceding comment)

Comment: 36: Consider rephrasing the term "behavioral factors" throughout the document, as it is not technically accurate. The study investigates the factors that influence behavior, many of which are not behavioral in nature (e.g., opportunities)

Response: Thanks so much for your suggestion. We have edited throughout the manuscript.

Comment: 39: Clarify gap: There is a large body of research on the factors that influence veterinarian behavior, however, there is a need for more studies that use comprehensive behavior change models to develop and evaluate interventions. This is the value this study adds from my perspective.

Response: Thanks so much for your suggestion. We have added this. Please check line number 38-40 in the revised version.

Comment: 107 – 110. There is a growing body of evidence that suggests that the constructs used in these models are not always reliable predictors of behavior. This is known as the intention-action gap, which is a well-studied phenomenon (including agricultural sciences). As a result, the theories mentioned here are no longer recommended.

Response: Thanks so much for your suggestion. We have removed it from the revised version.

Comment: 122-123: It is important to note that the COM-B model and the Behavior Change Wheel (BCW) are two different but complementary frameworks. While the COM-B model is specifically designed to investigate the factors that influence behavior, the BCW is a framework for identifying and selecting behavior change interventions. The authors may want to consider focusing on the COM-B model in the main body of the document, and mentioning the BCW in the discussions when recommendations for interventions are made. This is because the COM-B model is a more general framework for understanding the three essential components of behavior, while the BCW is a more specific framework for identifying and selecting interventions.

Response: Thanks so much for your suggestion. We have removed it from the revised version and introduce BCW as a guide for developing and selecting behavior change interventions in the discussion and recommendation section.

Comment: 125: This is how the TDF was created. The COM-B is based on 1) a US consensus meeting of behavioural theorists in 1991 and 2) principle of US criminal law.

Response: Thanks so much for the information. Yes, we are aware of it. Hence, we edited in the revised version. Please check 161-162 in the revised version.

Comment: 130: The authors may want to consider using the original definition of motivation (and the other COM-B domains) from the original paper. This definition is more inclusive of both reflective and automatic decision-making.

Response: We have made changes and added the original definition from the original paper with proper citation. Please check line number 167-168 in the revised version.

Comment: 133: The authors may want to clarify that the TDF is not built upon the COM-B model. In fact, the first version of the TDF was published in 2005, while the COM-B model was published in 2011 (both Michie et al.). However, the two frameworks are complementary, and the revised version of the TDF by Cane et al. (2012) shows how they can be combined to advance the level of detail and complexity.

Response: Thanks so much for pointing this out. We have now edited the text in the revised version, line number 170-171.

Comment: 133-136: The last two sentence could be simplified for readers not familiar with this.

Response: Agreed. We have simplified in the revised version. These sentences now read: Comprising 14 domains, the TDF offers a comprehensive assemble of the overlapping constructs within behavioral theories and provides explanation of each domain [42, 43].

Comment: 146: Also consider these studies from same authors:
- Farrell, S., Benson, T., McKernan, C., Regan, Á., Burrell, A. M., & Dean, M. (2023). Factors influencing dairy farmers' antibiotic use: An application of the COM-B model. Journal of Dairy Science, 106(6), 4059-4071.
- Farrell, S., Benson, T., McKernan, C., Regan, Á., Burrell, A., & Dean, M. Exploring Veterinarians’ Behavior Relating to Antibiotic Use on Dairy Farms Using the COM-B Model of Behaviour Change. Available at SSRN 4328183.

146: Additional review to consider: Biesheuvel, M. M., Santman-Berends, I. M., Barkema, H. W., Ritter, C., Berezowski, J., Guelbenzu, M., & Kaler, J. (2021). Understanding farmers' behavior and their decision-making process in the context of cattle diseases: a review of theories and approaches. Frontiers in veterinary science, 8, 687699.

Response: Thanks so much for your suggestion. The first two papers are same, one was a pre-print which was published. We cited that study (Farrell et al 2023) in the revised version.

Comment: 214: It would be helpful to clarify the distinct steps involved in developing the deductive and inductive codes.

Response: Thanks for your comment. The steps for creating the inductive codes were described in the original draft. Line number 212-216. The strategy for creating deductive codes was described in line number 2011-212 in the original draft.

Comment: 217: The authors should clarify if and how the TDF was used as an analytical framework in the paper. It is mentioned throughout, however, not mentioned here. Additionally, it is unclear how the different types of codes generated are used.

Response: Thanks so much for your observation. As we showed and mentioned in the conceptual framework section of the paper, the TDF and COM-B were integrated together based on the nature of their domains. The analytical approach for this study was also built on that integrated way. This approach is aligned with the papers that you suggested and referred in the earlier comment.

Comment: 239: Add reference (Cane et al. (2012)). Please clarify how the TDF was used as an analytical framework in the paper, or if it wasn’t used remove the TDF column. If the TDF was used, please specify which inductive codes were mapped to the TDF domains.

Response: Thanks for your comment. We think this is closely linked with the previous response, please see the previous response.

Comment: 273: self-efficacy. I suspect it is “perceived competence” but this needs a quote to conform. Additionally, the concept is not explicitly mentioned in the table above, but it could be classified under the TDF domain of "beliefs about capabilities" and the COM-B component of "reflective motivation."

Response: Thanks for your suggestion. We have updated with ‘perceived competence’ however, this statement was made based on the comparison on the pattern of text segments from the inductive analysis. Hence, we do not have specific quotation to confirm it. Please check 318 in the revised version.

Comment: 352: It may be helpful to clarify that the statement "participants believe that livestock owners focus on money and want to raise more poultry per land area to minimize costs while maximizing profit" represents the vets' beliefs, not necessarily reality.

Response: Thanks so much for your suggestion. We have edited in the revised version. Please check the line number 401-402 in the revised version.

Comment: 376: Avoid mentioning numbers in a qualitative study, unless they are essential to the findings or to provide context.

Response: We have removed the number.

Comment: 436: Clarify if this is what participants believe

Response: Clarified. This sentence now reads: One widespread perception among the veterinarians is that vets who do not suggest antibiotics are not good vets. Please check line number 488 in the revised version.

Comment: 460: Fear of losing job. Consider this moving to motivation either as emotion or as beliefe of consequences.

Response: Thanks for your suggestion. However, as we found this as part of discussing the (negative) influence by social actors, we would like to keep the placement as it is.

Comment: 467: …to promote reduced AMU/act as AMR stewards. Use the same behavior you are looking at throughout the paper.

Response: Thanks for your suggestion. We have kept the same behavior throughout.

Comment: 493: Please explain why the component described in this section is classified as "motivation," since this component describes internal processes that trigger or inhibit behaviour.

Response: Thanks for your question. We considered updating the treatment protocol and proper guidelines by the veterinary council as a reflective process (involving evaluation and plans). Hence, we classified this section under “Motivation”.

Comment: 520: Beliefs about impact. TDF term is beliefs about consequences

Response: Edited. This sentence now reads: Beliefs about consequences of reduced prescription of antimicrobials.

Comment: 535: Quote suggest that the fear of losing one's reputation due to unsuccessful treatment is the underlying driver of this behavior

Response: We have included in the revised version. Please check line number 589-590.

Comment: 560 – 564: Behaviors influenced by the actions or perceptions of others should be classified under the "social opportunity" domain.

Response: Thanks for the comment. This quotation is more on flow of the conversation and refers to the negative optimism or barrier related to optimism. We have very specific results in the opportunity section. Please see Social Opportunity Section to be specific.

Comment: 565-567: Please explain how lack of monitoring or law enforcement can influence motivation. It may also fall under external opportunities depending on the exact quote.

Response: Again, this is part of the factors related to their motivation. “If” there was proper monitoring or adequate law enforcement in place, vets would feel that “reduced prescription of antimicrobials by themselves” would work.

Comment: 569-570: elaborate why this is important or relevant.

Response: This is one of the contributions of this study to the existing literature. We have edited this sentence to read: To the best of our knowledge, this is the first study that has applied the COM-B model to explore factors behind veterinarians' antimicrobial prescribing behaviors in a LMIC.

Comment: 575: Inductive inference. Please consider if this is more appropriately conceived as deductive analysis

Response: Thanks for your comment. The findings under the individual domain of COM-B model are retrieved from the inductive analysis – this was mentioned in the analysis of the method section.

Comment: 609: consider rephrasing to “reduce the need for AMs

Response: Edited. Please check line number 664-665 in the revised version.

Comment: 674: The data analysis section does not mention thematic analysis. Also mention here, that both thematic analysis and framework analysis was used.

Response: In line 242 (first sentence of the Data Analysis section), we specifically mentioned that we conducted? thematic analysis in this study. As we used a framework, we used that framework as our guiding principle to carry out the thematic analysis by using both inductive and deductive codes.

Comment: 681: Please explain how the two frameworks were integrated into the data analysis

Response: This is mentioned in Figure 1 under the Conceptual Framework section and line number 250-258 in the data analysis section of the revised version. Explaining here would be redundant.

Comment: 699-701: I am not sure how the BCW can directly suggest this. A better approach might be to start with the broader policy (or intervention function) categories and then move on to specific suggestions

Response: Edited. This sentence now reads: Finally, veterinarians should focus on building strong relationships with farmers to reduce farmers’ likelihood to seek antimicrobials elsewhere, particularly from informal quacks. Please check line number 775-777 in the revised version.

Minor comments in the PDF File:

13.-1: AMR is broader than antibiotics and encompasses all antimicrobials. Suggest saying “antimicrobial use” or specifying that you are talking about antibiotic resistance.

Response: Revised the introduction section throughout.

13-3: Move references here.

Response: Moved the reference at the end of the sentence. Please check line number 68 in the revised version.

14-1: Provide an example here “influential relationships (e.g. with pharmaceutical companies).

Response: Added as suggested. Please check line number 116-117 in the revised version.

14-2: Better placed in Discussion unless you provide a citation.

Response: Added a reference. Please check line number 130 in the revised version.

20-1: participants should be lower case.

Response: Edited. Please check line number 264 in the revised version.

20-2: Would spell this out for non-vet readers.

Response: Spelled out. Please check line number 273 in the revised version.

21-1: What does freelance mean? Provide definition in caption or table footnote.

Response: Defined. Please check line number 274 in the revised version.

22-1: A stylistic note but would put capability in caps “CAPABILITY” to emphasize. Suggest doing the same for opportunity and motivation.

Response: Thanks so much for your suggestion. Edited as suggested. Please check throughout.

23-1: It will remain [in the] legs.

Response: Edited as suggested. Please check line number 301 in the revised version.

23-2: Suggest “the complete failure of medicines”

Response: Edited as suggested. Response: Edited as suggested. Please check line number 304-305 in the revised version.

23-3: Suggest “participants who received AMR training”

Response: Edited as suggested. Response: Edited as suggested. Please check line number 310 in the revised version.

24-1: Suggest “respective uses”

Response: Edited as suggested. Response: Edited as suggested. Please check line number 319 in the revised version.

25-1: Antivirals, which are antimicrobials, exist for FMD. Suggest rewording of “Antibiotics cannot directly target FMD but they….”

Response: Edited as suggested. Response: Edited as suggested. Please check line number 338-339 in the revised version.

26-1: Suggest replacing hospital with veterinary.

Response: Edited as suggested. Response: Edited as suggested. Please check line number 368 in the revised version.

26-2: Do you mean veterinarians and vet clinics?

Response: Yes, edited. Please check line number 368 in the revised version.

26-3: hassles

Response: Edited. Please check line number 369 in the revised version.

26-4: Comma after.

Response: Edited.

26-5: Do you mean “ruminants, such as goats” given goats are ruminants?

Response: yes.

26-6: and AMR outside of connection with AMU.

Response: Edited. Please check line number 381 in the revised version.

27-1: “ensuring a”

Response: Edited. Please check line number 382 in the revised version.

28-1: Recently administered to that specific animal?

Response: Yes, edited. Please check line number 419 in the revised version.

28-2: Suggest defining what this means in brackets

Response: Defined. Please check line number 426-427 in the revised version.

29-1: Gumboro

Response: Edited. Please check line number 434 in the revised version.

29-2 antibiotic.

Response: edited. Please check line number 435 in the revised version.

29-3: Do you mean antibiotics specifically?

Response: No, this is antimicrobials in general, that includes antibiotics.

30-1: Suggest “The veterinarians indicated that farmers are not aware…”

Response: Edited as suggested. Please check line number 464-465 in the revised version.

31-1: They are talking about antimicrobials as antibiotics an important point

Response: In our observation, participants sometimes intentionally mentioned antimicrobials on some occasions while they used antibiotics as well. On most of the cases, they used either term unintentionally. Hence, we kept the original text segment in the quotation.

42-1: Suggest a subheading here of “Study Limitations”

Response: Added. Please check line number 723 in the revised version.

43-1: Replace with “participated”

Response: Edited as suggested. Please check line number 779 in the revised version.

Reviewer #2: The idea behind the study is great and presents useful information on the factors influencing antimicrobial prescription behaviours of Vets. However, the authors have not done justice to the paper especially given the nature of the framing of the paper especially at the presentation of results and subsequently the discussion. There is need to properly link the findings to the results from the informants and to the components of the COM-B Model

Response: Thank you for your comment and observation. We want to clarify that our study adheres to the COM-B model as our primary study and analytical framework. Notably, the results section is structured according to the domains of the COM-B model, and similarly, the discussion section is crafted based on our findings, which are then compared and discussed in relation to previous studies. We are open to making adjustments and edits to the paper, especially if specific issues regarding the presentation of findings and the organization of the discussion section are highlighted. Providing specific and explicit comments will greatly assist us in enhancing the clarity of the manuscript.

Comment: There is need to check the whole document for grammatical errors especially the quotes which need to be edited for grammar and sentence structure.

Response: Thanks so much for your comment. The last author of this paper is a native English speaker who checked and reviewed the grammar. Additionally, the draft was reviewed by another native speaker who is a PhD student. However, we have checked and update the English where applicable.

Comment: Line 81-83- Is the problem of misuse and abuse linked to prescription by vets or self-medication?
Response: Thanks for your question. We specified here “over-the-counter sale” accelerates misuse or overuse of antimicrobial – which is one of the main reasons for self-medication. Please check line number 93 in the revised version.

RESULTS

Comment: Summary 1 Table 1 page 13. How was knowledge defined as fair or lacking?
Specific comments include: There is need to indicate how knowledge was measured and what entailed fair knowlege.
Was knowledge equated with awareness?

Response: Thanks for your question. Fair knowledge was framed when the participants were able to make references and explain core concepts in this field such as the concept of “antimicrobial resistance”, “reserve group of antibiotics” “differentiate different groups of antimicrobials and their use” “broad and narrow spectrum” “applicability of antimicrobials”.

No, knowledge was not equated with awareness.

Comment: Line 276- It is not clear whether the quote from line 277-282 is from an experienced or junior vet.
Response: This quote was retrieved from an interview with a vet who had 12 years of experience. Please check line number 323 in the revised version.

Comment: It is not clear whether the motivation is that vets are fearful about failure of treatment. How does this point link to influencing the Vets behaviour? There needs to be a clear linkage of this motivation

Response: Thanks for your comment. From the article on COM-B model, motivation is defined as all those brain processes that energize and direct behavior, not just goals and conscious decision-making [Please see PMID: 21513547]. It includes habitual processes, emotional responding, as well as analytical decision-making. This adds non-volitional mechanisms involved in motivation (e.g., habits) and to conceptualize causal associations between the components in an interacting system.

The quotation we used in the case of fear of treatment failure related to non-volitional motivation because they felt that treatment failure could occur with some basic medicine (BUT not prescribing antimicrobials). Hence, beliefs on the impact of reducing antimicrobial prescription have mediate the causal association between non-volitional motivation and behavior.

Comment: Line 301 seems to be hanging
Response: Edited. Please check line number 349 in the revised version.

Comment: Under sub topic 4.2.2 Line 306 – 307 is framed more like a barrier as opposed to an opportunity…. Generally the framing of the results need to bring out the key components under the COM-B Model eg the opportunities sound more like barriers, it would be great to frame them as opportunities that vets take advantage of to prescribe antibiotics.
It is not clear whether the opportunities influences negatively or positively , This needs to come out clearly – How is environmental factors an opportunity and is it an opportunity for antimicrobial prescription behaviour of a farmer?

Line 322-328: As a result, farmers 324 feel reluctant to bring their large animals (for example ruminants or goats) due to the transportation325 hassle and cost. One participant said,
326 “……this is particularly a huge barrier for the farmers those who have cows or goats due

327 to the long distance and cost. Because farmers have to hire a transportation like motorized

Response: Thanks for your comment. In the COM-B model, opportunity defines all the factors that lie outside the individual that make the behavior possible or prompt it. Opportunity is distinguished between physical opportunity afforded by the environment and social opportunity afforded by the cultural milieu that dictates the way that we think about things (e.g., the words and concepts that make up our language). However, limitations on the sub-domains of the opportunity domain are also categorized under opportunity, that are mainly the barriers related to this domain. Please see details in the original article where COM-B model is explained [PMID: 21513547]. In our study, we found and discussed both social and environment factors. Given the example of the comment, this is an issue with opportunity because farmers’ reluctance and problems with transporting [due to the absence of physical opportunity] the animals to the vets influence vets’ decision to prescribe antimicrobials. In this case, they ‘have to' prescribe due to the lack of opportunity.

Comment: 328 van which costs a lot of money.” (Participant – 12). The authors have not linked this well with the opportunity for vets to reduce prescription of antimicrobials.

Response: Thanks so much for catching it. We have now linked it to the opportunity. Please check line number 377-379 in the revised version.

Comment: Under line 331-335 – Do the authors mean animal management of is it farm management? From my perspective farm management is about the infrastructure of the farm.
Authors need to clearly relate the aspects of the facilities related to farm management and biosecurity to veterinarians’ behaviour in relation to antimicrobial prescription.

Response: Thanks for your question. By saying “farm management” broadly, we meant to refer to both aspects including proper management of farms and animals. In our findings, we included issues related to animal management such as feeding, immunization, and nutrition for the animals, as well as farm management such as biosecurity of the farm and flock size.

In response to your question about how biosecurity and facilities relate to farm management, we have specifically mentioned that improper management and lack of biosecurity increased the risk of bacterial and viral infections. In this context vets mentioned that the use/prescription of antimicrobials increase when there is high risk of growing infections under such conditions. This is described in lines 389-394.

Comment: Its important to bring out the voices of the informants. In the text it is sometimes not easy to differentiate the voices of the authors from those of the informants eg
Line 467 sub title: 4.2.3 Veterinarians’ motivation to promote reduced AMU/act as AMR stewards
The text under this sub title indicate what the vets would love to see but the authors could consider outlining and presenting information in relation to what t motivates them to promote reduced AMY/act as Stewards

Response: Thanks so much for your observation. However, the findings we presented under this theme are based on the COM-B model – as this is our conceptual and analytical framework. For the presentation of the findings, we made statements based on the ‘thick description’ approach (as referred by Hennink, Hutter and Bailey in their book “Qualitative Research Methods”) and we presented quotations to conform our statements under each themes and sub-themes.

Comment: In some situations the authors talk about farmer behaviour. It is not clear how the farmers behaviour is linked to the vets behaviour in relation to prescribing antimicrobials.

Response: Thanks for your comment. In our study the vets do not operate alone and hence their prescription behavior is influenced by different actors including the farmers. Where applicable, in our result section, we have outlined how farmers’ behavior influence vets’ behavior. Specifically, in the “Influence of farmers” sub-section under the social influence section, we have presented findings on how farmers’ behavior influence vets to prescribe antimicrobials. Some examples included farmers’ reliance on informal service providers, farmers’ reluctance to bring the animals to the vets, farmers incompliance in following recommendations to determine flock size etc.

Comment: How have the socio-demographic aspects of the informants been weaved into the presentation of the results?

Response: Thanks for your question. The main purpose of presenting the socio-demographic aspects was to provide background information about the participants. We also compared the findings across the demographic groups such as years of experience, level of formal education, location of practice, and organizational affiliation. The comparisons were reported when we found differences.

6. PLOS authors have the option to publish the peer review history of their article ([what does this mean?](https://journals.plos.org/plosone/s/editorial-and-peer-review-process#loc-peer-review-history)). If published, this will include your full peer review and any attached files.

If you choose “no”, your identity will remain anonymous but your review may still be made public.

**Do you want your identity to be public for this peer review?** For information about this choice, including consent withdrawal, please see our [Privacy Policy](https://www.plos.org/privacy-policy).

Reviewer #1: No

Reviewer #2: No

[NOTE: If reviewer comments were submitted as an attachment file, they will be attached to this email and accessible via the submission site. Please log into your account, locate the manuscript record, and check for the action link "View Attachments". If this link does not appear, there are no attachment files.]

While revising your submission, please upload your figure files to the Preflight Analysis and Conversion Engine (PACE) digital diagnostic tool, <https://pacev2.apexcovantage.com/>. PACE helps ensure that figures meet PLOS requirements. To use PACE, you must first register as a user. Registration is free. Then, login and navigate to the UPLOAD tab, where you will find detailed instructions on how to use the tool. If you encounter any issues or have any questions when using PACE, please email PLOS at [figures@plos.org](mailto:figures@plos.org). Please note that Supporting Information files do not need this step.

*In compliance with data protection regulations, you may request that we remove your personal registration details at any time.* [*(Remove my information/details)*](https://www.editorialmanager.com/pone/login.asp?a=r)*. Please contact the publication office if you have any questions.*
