# Supplementary material for: An application of COM-b model to explore factors influencing veterinarians’ antimicrobial prescription behaviors: Findings from a qualitative study in Bangladesh
Source: PLoS One. 2024 Dec 16;19(12):e0315246. doi: 10.1371/journal.pone.0315246 (PMC11649135; doi:10.1371/journal.pone.0315246)
Supplement: Attachment — Submitted filename: Response to editor.docx [file pone.0315246.s004.docx]

Comment: It looks like the concept of selection of resistance by the participants is not always known (L297), in next paragraph some other participants correctly use the concept of selection of resistance, though the example given states antibiotic resistance will be created in the human body, which indicates that this participant does not know how resistance evolves.

Response: Thanks for your comment and identify the nuance. However, given the different pathways of growing AMR, our participants are aware how AMR evolves and provided these two explanations. Regarding to the spread of AMR to human, that specific participant meant how consumption of chicken may lead to human AMR. This is an example. That means, this another way to grow AMR. This is an awareness related both animal and human AMR. However, we have edited the texts to increase the clarity. Please check line number 248-253 in the revised version.

Comment: L308 speaks again over development of AMR, while it should be selection of AMR. Discuss this and use correct terminology.

Response: Thanks so much for your comment. This section is about participants’ knowledge on AMR and how it develops. However, in the consecutive sub-sections of the result section, we have outlined the other drivers that influence the selection of antimicrobials. We have edited the language for the clarity. Please check line number 263 to 264 in the revised version.

Comment: Participant 14, L328, shows another problem and that is that penicillin is not effective against the bacteria listed… The participant does not know what antibiotics to use for which infection.

This indicates that there are other gaps in the knowledge. Please address

Response: Thanks so much for pointing this out. We were looking back at our transcript. We think there was a typo during the translation phase. Basically, the participant mentioned “Ciprofloxacin” which is now edited and updated. Please check the line number 282 in the revised version.

Comment: L734 Companion animals and pets are the same, they are moreover domestic, adapt

Response: Thanks for your catching this. We have edited in the revised version. Please check the line number 677 in the revised version.
