# Supplementary material for: An application of COM-b model to explore factors influencing veterinarians’ antimicrobial prescription behaviors: Findings from a qualitative study in Bangladesh
Source: PLoS One. 2024 Dec 16;19(12):e0315246. doi: 10.1371/journal.pone.0315246 (PMC11649135; doi:10.1371/journal.pone.0315246)
Supplement: Attachment — Submitted filename: Reviwers comments24102024.docx [file pone.0315246.s005.docx]

Thank you for the opportunity to review this manuscript. It presents a compelling and important study that explores the behavioural factors influencing antimicrobial prescription practices among registered veterinarians in Bangladesh. The authors have made a significant effort in addressing the feedback from both reviewers, and the manuscript demonstrates several strengths that warrant its acceptance for publication.

Here is a summary of the key reasons for my recommendation:

1. Relevance and impact: The topic of antimicrobial resistance and prescription behaviours among veterinarians is highly relevant, especially in low- and middle-income countries like Bangladesh. The manuscript addresses an urgent global health concern with a novel application of the COM-B model.
2. Robust theoretical framework: The authors have effectively used the COM-B model and Theoretical Domains Framework, which strengthens the manuscript’s contribution to the field of behaviour change in antimicrobial stewardship. The study is well-grounded in behavioural science, which adds depth to the findings.
3. Methodological rigor: The qualitative approach is sound, and the authors have provided clear explanations of their coding processes and thematic analysis. The use of both deductive and inductive methods ensures a rich dataset that aligns well with the research objectives.
4. Revisions based on reviewers’ feedback: The authors have responded appropriately to reviewers’ concerns. They have clarified terminology, corrected minor errors, and enhanced the manuscript’s clarity and precision. The response to the feedback shows a high level of engagement to improving the manuscript.

Conclusion:

With the effort the authors have put into addressing the reviewers' comments, the manuscript demonstrates sufficient rigor and relevance for publication. Given the timely and well-executed nature of the research, I would recommend acceptance, especially with the improvements made after the review process. The study's contribution to AMS practices in veterinary medicine, particularly in resource-limited settings, is valuable and adds to the growing body of global health literature.
