# Supplementary material for: An application of COM-b model to explore factors influencing veterinarians’ antimicrobial prescription behaviors: Findings from a qualitative study in Bangladesh
Source: PLoS One. 2024 Dec 16;19(12):e0315246. doi: 10.1371/journal.pone.0315246 (PMC11649135; doi:10.1371/journal.pone.0315246)
Supplement: Attachment — Submitted filename: Reviewers comments and our responses.docx [file pone.0315246.s006.docx]

**Comments to the Author**

1. If the authors have adequately addressed your comments raised in a previous round of review and you feel that this manuscript is now acceptable for publication, you may indicate that here to bypass the “Comments to the Author” section, enter your conflict of interest statement in the “Confidential to Editor” section, and submit your "Accept" recommendation.

Reviewer #3: All comments have been addressed

Reviewer #4: (No Response)

Reviewer #5: All comments have been addressed

Reviewer #6: All comments have been addressed

Reviewer #7: All comments have been addressed

Reviewer #8: (No Response)

Reviewer #9: All comments have been addressed

Reviewer #10: (No Response)

2. Is the manuscript technically sound, and do the data support the conclusions?

The manuscript must describe a technically sound piece of scientific research with data that supports the conclusions. Experiments must have been conducted rigorously, with appropriate controls, replication, and sample sizes. The conclusions must be drawn appropriately based on the data presented.

Reviewer #3: Yes

Reviewer #4: Yes

Reviewer #5: Yes

Reviewer #6: Yes

Reviewer #7: Partly

Reviewer #8: Yes

Reviewer #9: Yes

Reviewer #10: Yes

3. Has the statistical analysis been performed appropriately and rigorously?

Reviewer #3: Yes

Reviewer #4: I Don't Know

Reviewer #5: N/A

Reviewer #6: I Don't Know

Reviewer #7: N/A

Reviewer #8: No

Reviewer #9: Yes

Reviewer #10: I Don't Know

4. Have the authors made all data underlying the findings in their manuscript fully available?

The [PLOS Data policy](http://track.editorialmanager.com/CL0/http:%2F%2Fwww.plosone.org%2Fstatic%2Fpolicies.action%23sharing/1/010f019302998a03-bd305bab-1b6e-4e5e-811c-9bd5c46bc75f-000000/P6ttm_WG8B7kbl-g8lNFtTvVFhi7mzOR4tlFRj58znQ=184) requires authors to make all data underlying the findings described in their manuscript fully available without restriction, with rare exception (please refer to the Data Availability Statement in the manuscript PDF file). The data should be provided as part of the manuscript or its supporting information, or deposited to a public repository. For example, in addition to summary statistics, the data points behind means, medians and variance measures should be available. If there are restrictions on publicly sharing data—e.g. participant privacy or use of data from a third party—those must be specified.

Reviewer #3: Yes

Reviewer #4: No

Reviewer #5: No

Reviewer #6: Yes

Reviewer #7: Yes

Reviewer #8: No

Reviewer #9: Yes

Reviewer #10: Yes

5. Is the manuscript presented in an intelligible fashion and written in standard English?

PLOS ONE does not copyedit accepted manuscripts, so the language in submitted articles must be clear, correct, and unambiguous. Any typographical or grammatical errors should be corrected at revision, so please note any specific errors here.

Reviewer #3: Yes

Reviewer #4: Yes

Reviewer #5: Yes

Reviewer #6: Yes

Reviewer #7: Yes

Reviewer #8: Yes

Reviewer #9: Yes

Reviewer #10: Yes

6. Review Comments to the Author

Please use the space provided to explain your answers to the questions above. You may also include additional comments for the author, including concerns about dual publication, research ethics, or publication ethics. (Please upload your review as an attachment if it exceeds 20,000 characters)

Reviewer #3: Comments raised by the reviewers has been addressed satisfactorily by the authors, the manuscript is technically and scientifically sound, the content can contribute immensely to the field of antimicrobial resistance.

Response: Thanks so much for reading the manuscript and sharing the encouraging observation.

Reviewer #4:

Comment: The research article entitled “An Application of COM-B Model to Explore Factors Influencing Veterinarians’ Antimicrobial Prescription Behaviors: Findings from a Qualitative Study in Bangladesh” has been reviewed. In the study, Shano and co-workers applied the Capability, Opportunity and Motivation for Behaviour (COM-B) model to comprehensively study the behavioural change models to evaluate antimicrobial prescriptive interventions among registered veterinarians in Bangladesh. The study mainly involved one-on-one interviews with 32 participants from the aforementioned professionals. Positively, it could have impacted the policy makers on necessary regulations on the prescription behaviours of antimicrobial prescriptions by veterinarians in Bangladesh and some other nations affected by the knowledge covered. However, most of the highlighted outcomes are ideally expected to have been undertaken by the professionals as important parts of curriculum during the course of training. If so, then what is the significance of this study? Authors need to strongly indicate that the new findings are not already existing knowledge in the course of training professional veterinarians that prescribe antimicrobials or channel their efforts towards the implementation of the known knowledge/policy. Based on this and other comments enlisted below, I recommend a major revision before the manuscript can be accepted for publication in this title.

Response: Thank you for your comment. However, we respectfully disagree with your interpretation of our paper and its merit for several reasons.

Firstly, the veterinary curriculum does not typically include the COM-B model to enhance knowledge. The COM-B model is a behavioral framework applicable to a wide range of health behaviors, beyond just knowledge improvement.

Secondly, our study goes beyond assessing psychological capability, knowledge, or training-related factors. We also examine critical elements such as physical opportunity (e.g., access to testing facilities, environmental context, structural barriers), social opportunity (e.g., social influences, peer pressure, norms), and factors related to negative reflective motivation. Notably, even with substantial knowledge and training on AMR, these additional factors limit participants' ability to prescribe antimicrobials appropriately.

Thirdly, our recommendations extend beyond training and education. We advocate for a comprehensive strategy that includes enhancing physical opportunities and engaging stakeholders.

Finally, our study is one of the few to use the COM-B model as a conceptual framework in low- and middle-income countries. Therefore, the findings of this study are a novel contribution to the existing literature.

However, as detailed below, we have addressed your remaining comments.

Comment 1: 1. Understandably, the cause of antimicrobial resistance extends beyond an excessive usage but also importantly entails misuse of antimicrobials and several other factors including poor hygiene, lack of sanitation, contamination of food, drink and environments, inadequate preventive and control measures etc. How have the authors considered the influence of these other factors on the prescription behaviour of veterinarians? In addition, evolvement of resistance mechanisms towards the existing medications oftentimes constitute an increase in antimicrobials prescription. Authors need to also consider this in their recommendations.
Response: Thank you for your comment. The primary focus of our study was to understand the prescribing behavior of veterinarians and the factors that influence their decisions to prescribe antimicrobials. While the factors you mentioned are indeed critical in studying AMR, including all of them in a single study would have added considerable complexity and required a different study design. Furthermore, many of these factors have already been explored in the Bangladeshi context by other researchers.

However, our participants did highlight several relevant issues. Based on their responses, we reported factors such as farm management, infection prevention and control, hygiene practices, and the impact of climate change on antimicrobial prescribing. Therefore, our recommendations include strategies aimed at enhancing these specific areas.

Comment: 2. Were the COM-B practices not already in the curriculum or courses of studies undertaken by the formal professionals during their trainings? If yes, what is the significance of this present study? Perhaps the results from this study could discourage.

Response: No. COM-B is a behavioral model that can be applied in identifying behavioral factors for any health behaviors. Please see the paper about COM-B model for details: <https://implementationscience.biomedcentral.com/articles/10.1186/1748-5908-6-42>

Comment: 3. Authors are advised to minimize the usage of personalized statements and instead, adopt reporting formats. E.g. use “the study was conducted by…” instead of “we conducted the study by…”

Response: Edited as suggested. Please check page 10, line, 184-185 in the revised version.

Comment: 4. What influenced the significant gender bias in authors’ criteria for participant selections and how could these not affect the fairness in the outcomes?

Response: Thank you for your comment. Firstly, women's participation in veterinary education is significantly lower than men's, due to various social and cultural factors. As a result, we observed that only a small number of women are registered in veterinary practice, and their representation in the field is very limited. During our recruitment process, we made efforts to include as many women veterinarians as possible. However, we were unable to increase their numbers due to structural and systemic barriers beyond our control.

Comment: 5. In section 3.2, the authors have mentioned bachelor degree holders among the recruited participants. However, this class is missing in the table of participant’s characteristics (Table 1). Kindly clarify.

Response: Thanks for your comment. This category includes with DVM (Doctor of Veterinary Medicine). We have included this in the parenthesis in page 13, line 231, in the revised version.

Comment: 6. I strongly advise that the knowledge depths of the selected participants should also be considered to validate the results from this study. For instance, if a farmer intends to sell his chickens on 30th and they developed microbial infection on 27th as mentioned by a participant, what should be done by the farmer for his business security and human safety?

Response: Thanks for your comment. The example given by the participant was about their knowledge about describing AMR and its development. We are not validating their knowledge, but a presentation how they explained AMR. This will ultimately reflect on the need of accurate knowledge through either training or workshop as part of antimicrobial stewardship initiative.

Comment: 7. Were the quantitative data from this study analyzed statistically? Kindly indicate the location if available.

Response: This is a qualitative study, not quantitative. Hence, we do not have any quantitative data or statistical analysis.

Comment: 8. The English language of this submission requires a thorough recheck by an expert. Authors should consider this.

Response: This manuscript was read by a native individual who independently checked grammar and English. Additionally, one of the authors (senior author) is a native English speaker who also checked and improved English during the previous rounds of resubmission.

Reviewer #5: (No Response)

Reviewer #6: The previous comments have been addressed. However, in responses, Line 281 it is mentioned that ciprofloxacin is narrow spectrum antibiotic. Kindly check the transcript again or otherwise this still shows some knowledge gaps as ciprofloxacin is broad spectrum.

Response: Thanks so much for your concern. We have checked the audio recording and transcript of that interview. We have now corrected with ‘broad’ in the revised manuscript. Please check page 16, line number 281 in the revised version.

Reviewer #7: Both reviewers are well explained each of the statement. Its an interesting survey work. But one things, Its not a clear idea how antibiotic resistance created human body after eating poultry products?

Response: thanks for your comment. The relative explanations are participants reasoning on AMR. As we mentioned in the manuscript, participants used a variety of explanations, and identified several pathways including the respecting the withdrawal periods. In the case of chicken, for example one quotation (page 15, lines 255-260) specifically indicated, if the withdrawal period is not respected, administering antibiotics to poultry shortly before slaughter can lead to antibiotic residues in the meat. When humans consume this meat, it contributes to the development of antibiotic-resistant bacteria in their bodies.

Reviewer #8: Dear authors, I have the following concerns
Comment 1. The sample size (32) is too small to draw strong evidence to fulfil the objectives. according to the Bangladesh Veterinary Council, the country has more than eight thousand veterinarians, then how these 32 will present the other. This was also expressed under the limitation. However, it is too small to fulfil objectives.

Response: Thank you for your comment regarding the sample size. We respectfully disagree that a sample size of 32 interviews is too small for our study. We believe the sample size was appropriate based on the following qualitative research principles.

Firstly, saturation achieved: In qualitative research, the goal is not to generalize to the entire population but to achieve saturation, where no new themes or insights emerge from additional data collection. In our study, we reached saturation after 32 interviews, indicating that the sample size was sufficient to capture the breadth and depth of relevant themes.

Secondly, diverse sampling for rich data: We ensured a diverse and purposive sampling strategy by including participants from various backgrounds, including different genders, years of practice (experience), geographic locations (urban vs. rural), nature of affiliations (government vs. non-government), and educational qualifications (bachelor’s, master’s, or higher degrees). This diversity in sampling enhances the transferability of our findings by capturing a wide range of perspectives, making our results more robust.

Thirdly, credibility and reliability of findings: Our methodological approach adhered to principles of credibility and reliability in qualitative research. We used a well-structured interview guide, conducted interviews until no new information was gained (saturation), and employed strategies such as member checking and triangulation to ensure the accuracy and consistency of our findings.

Fourthly, qualitative research purpose: The purpose of qualitative research is to explore and understand complex phenomena in-depth rather than to generalize to a larger population. Our focus was on understanding the nuances of veterinarians’ prescribing behaviors and the factors influencing them. Given this exploratory nature, a smaller, carefully selected sample is not only adequate but also consistent with qualitative research principles.

Finally, acknowledge and address limitations: While we acknowledge the limitation regarding the sample size in relation to the larger population of veterinarians, we addressed this in our discussion. The intent of our study was not to provide statistical generalizations but to gain in-depth insights into prescribing behaviors, which are best captured through qualitative methods.

Comment 2. Line 153, remove phrases like ----please see, put only something in the for further reference.

Response: Removed as suggested. Please check line number 153 in the revised version.

Comment 3. Line 255, instead of saying “some of the participants” why don't you write the number and/or of participants who answered that question. This is true throughout the document including the abstract

Response: Thank you for your comment and suggestion. However, we would like to clarify that the primary purpose of qualitative research is not to quantify the number of participants who expressed a particular view, but rather to provide in-depth insights, thoughts, and themes that emerge from the data. Using phrases like "some of the participants" is intentional, as it aligns with qualitative research principles, which focus on understanding the richness and complexity of the participants' experiences rather than providing numeric representations.

Counting the number of participants who mentioned a specific theme may give a false impression of quantitative generalizability, which is not the aim of qualitative research. Instead, our approach is designed to capture the diversity of perspectives and emphasize the contextual meaning behind the responses. We believe this method offers a more accurate and nuanced understanding of the data.

We hope this explanation clarifies our approach, and we appreciate your feedback on enhancing the clarity of our reporting.

Comment 4. Line 313 ------- Almost all the participants (n=24/32), this is 75%, not almost all... hence the sentence is not accurate.

Response: Thank you for your suggestion. Like we responded to the previous comment, in qualitative research, the focus is not on quantifying how many participants expressed a particular view, but rather on providing in-depth insights, themes, and diverse perspectives. Reporting numbers may create a false sense of quantitative generalizability, which is not the purpose of qualitative analysis. Instead, phrases like "some participants" help capture the richness and complexity of their experiences. We believe this approach aligns better with qualitative research principles and offers a more nuanced understanding of the findings.

However, we have now removed the number from the revised version.

Comment 5. Keywords are absent in the main part of the manuscript just after the abstract. There is the list in the submission system ---------◊ Global Health, One Health, AMR, Infectious Disease, Antimicrobial Prescription, COMB Model, Theory of Domain Framework, Behavior Change Wheel, Qualitative, Thematic Analysis, Veterinarians, Veterinary Practices, Bangladesh.

a. The list is too much

Response: Thanks so much for your comment. Inclusion of keywords were optional (please see PLOS Guidelines here: <https://journals.plos.org/plosone/s/submit-now>). However, we have now added a reduced number of key words to the main manuscript. Please check the line number 62-63 in the revised version.

Comment b. Global Health, One Health, Infectious Disease, Theory of Domain Framework, Wheel, and Thematic Analysis are not described well in the manuscript, please cancel

Response: Thanks so much for your comment. Generally, the keywords are used to make the papers searchable or findable. For PLOS One, keywords are used to help expedite processing of the manuscript. Hence, every keyword not necessarily need to well described in the manuscript. However, where applicable, we provided the details of the key words. For example, Theory of Domain Framework was described line number 133-135 and figure 1. They Keywords including Global Health, One Health and Infectious Disease are the broader umbrella of this paper. Hence, they do not need an explanation.

Comment c. Please don’t abbreviate the keywords, and separate by semi-colon

Response: As suggested, we have now added a reduced number of key words to the main manuscript. Please check the line number 62-63 in the revised version.

Comment 6. The conclusion was not presented separately and clearly

Response: Thanks so much for your comment. According to the journal guidelines, a conclusion was optional. Hence, we do not have a conclusion. However, since this study aimed at identifying the factors associated with antimicrobial prescribing behavior among registered veterinarians in Bangladesh through an implementation science framework, we provided a set a actionable recommendations.

Reviewer #9: Thank you for the opportunity to review this manuscript. It presents a compelling and important study that explores the behavioural factors influencing antimicrobial prescription practices among registered veterinarians in Bangladesh. The authors have made a significant effort in addressing the feedback from both reviewers, and the manuscript demonstrates several strengths that warrant its acceptance for publication.
Here is a summary of the key reasons for my recommendation:
1. Relevance and impact: The topic of antimicrobial resistance and prescription behaviours among veterinarians is highly relevant, especially in low- and middle-income countries like Bangladesh. The manuscript addresses an urgent global health concern with a novel application of the COM-B model.
2. Robust theoretical framework: The authors have effectively used the COM-B model and Theoretical Domains Framework, which strengthens the manuscript’s contribution to the field of behaviour change in antimicrobial stewardship. The study is well-grounded in behavioural science, which adds depth to the findings.
3. Methodological rigor: The qualitative approach is sound, and the authors have provided clear explanations of their coding processes and thematic analysis. The use of both deductive and inductive methods ensures a rich dataset that aligns well with the research objectives.
4. Revisions based on reviewers’ feedback: The authors have responded appropriately to reviewers’ concerns. They have clarified terminology, corrected minor errors, and enhanced the manuscript’s clarity and precision. The response to the feedback shows a high level of engagement to improving the manuscript.
Conclusion:
With the effort the authors have put into addressing the reviewers' comments, the manuscript demonstrates sufficient rigor and relevance for publication. Given the timely and well-executed nature of the research, I would recommend acceptance, especially with the improvements made after the review process. The study's contribution to AMS practices in veterinary medicine, particularly in resource-limited settings, is valuable and adds to the growing body of global health literature.

Response: Thank you very much for taking the time to read our manuscript and carefully evaluating its technical rigor and the knowledge gap it addresses. We appreciate your confirmation that we have adequately responded to the comments and suggestions from the previous reviewers. We also thank you for recommending the acceptance of our manuscript.

Reviewer #10:

Comment 1: L-42 semi-structured

Response: thanks for your suggestion. Updated with semi-structure. Please check line number 42 in the revised version.

Comment 2: L-63 as an invisible pandemic

Response: Updated as suggested. Please check line number 65 in the revised version.

Comment 3: L-70 lower-and-middle-income countries

Response: Updated as suggested. Please check line number 72 in the revised version.

Comment 4: L-71 as in the case of other LMICs

Response: Updated as suggested. Please check line number 73 in the revised version.

Comment 5: L-81 As in

Response: Updated as suggested. Please check line number 84 in the revised version.

Comment 6: L-82 the case of human antibiotics in Bangladesh

Response: Updated as suggested. Please check line number 84 in the revised version.

Comment 7: L-101 main behavioral factors that globally influence antimicrobials
Response: Updated as suggested. Please check line number 104 in the revised version.

Comment 8: L-114 behavior of veterinarians

Response: Updated as suggested. Please check line number 116 in the revised version.

Comment 9: L-121 We employed the Capability, Opportunity, Motivation and Behavior (COM-B)

Response: Updated as suggested. Please check line number 123 in the revised version.

Comment 10: L-126 The term ‘Capability’ refers to an

Response: Updated as suggested. Please check line number 128 in the revised version.

Comment 11: L-128 ‘Motivation’ denotes the brain

Response: Updated as suggested. Please check line number 130 in the revised version.

Comment 12: L-137 Conceptual framework: Integration of Theoretical Domains Framework (TDF) into the

Response: Updated as suggested. Please check line number 139 in the revised version.

7. PLOS authors have the option to publish the peer review history of their article ([what does this mean?](https://track.editorialmanager.com/CL0/https:%2F%2Fjournals.plos.org%2Fplosone%2Fs%2Feditorial-and-peer-review-process%23loc-peer-review-history/1/010f019302998a03-bd305bab-1b6e-4e5e-811c-9bd5c46bc75f-000000/_Sw1ypg8jVPql0r8z6mAT14Tx03n5nLkEQEmBg-2o-w=184)). If published, this will include your full peer review and any attached files.


If you choose “no”, your identity will remain anonymous but your review may still be made public.


**Do you want your identity to be public for this peer review?** For information about this choice, including consent withdrawal, please see our [Privacy Policy](https://track.editorialmanager.com/CL0/https:%2F%2Fwww.plos.org%2Fprivacy-policy/1/010f019302998a03-bd305bab-1b6e-4e5e-811c-9bd5c46bc75f-000000/pVPD4VhkykjFiEZKAPXnqAxroZ-eKhpefALgAa46Jbk=184).

Reviewer #3: **Yes:** Salim Faruk Bashir

Reviewer #4: No

Reviewer #5: No

Reviewer #6: No

Reviewer #7: No

Reviewer #8: No

Reviewer #9: **Yes:** Muhammad Augie Bashar

Reviewer #10: No
